# Vimentin Diversity in Health and Disease

**DOI:** 10.3390/cells7100147

**Published:** 2018-09-21

**Authors:** Frida Danielsson, McKenzie Kirsten Peterson, Helena Caldeira Araújo, Franziska Lautenschläger, Annica Karin Britt Gad

**Affiliations:** 1 Science for Life Laboratory, Royal Institute of Technology, 17165 Stockholm, Sweden; fridad@kth.se; 2 Department of Pathology, University of Utah School of Medicine, 84112 Salt Lake City, Utah, USA; mckenpeter@gmail.com; 3 Centro de Química da Madeira, Universidade da Madeira, 9020105 Funchal, Portugal; helena.caldeira@staff.uma.pt; 4 Campus D2 2, Leibniz-Institut für Neue Materialien gGmbH (INM) and Experimental Physics, NT Faculty, E 2 6, Saarland University, 66123 Saarbrücken, Germany; franziska.lautenschlaeger@leibniz-inm.de; 5 Department of Medical Biochemistry and Microbiology, Uppsala University, 75237 Uppsala, Sweden

**Keywords:** vimentin, intermediate filaments, cell‒extracellular matrix adhesions, extracellular vimentin, cell mechanical stiffness or elasticity, cellular contractility, biomechanics, epithelial‒mesenchymal transition, tissue regeneration, cancer and metastasis, drug target, clinical biomarkers

## Abstract

Vimentin is a protein that has been linked to a large variety of pathophysiological conditions, including cataracts, Crohn’s disease, rheumatoid arthritis, HIV and cancer. Vimentin has also been shown to regulate a wide spectrum of basic cellular functions. In cells, vimentin assembles into a network of filaments that spans the cytoplasm. It can also be found in smaller, non-filamentous forms that can localise both within cells and within the extracellular microenvironment. The vimentin structure can be altered by subunit exchange, cleavage into different sizes, re-annealing, post-translational modifications and interacting proteins. Together with the observation that different domains of vimentin might have evolved under different selection pressures that defined distinct biological functions for different parts of the protein, the many diverse variants of vimentin might be the cause of its functional diversity. A number of review articles have focussed on the biology and medical aspects of intermediate filament proteins without particular commitment to vimentin, and other reviews have focussed on intermediate filaments in an in vitro context. In contrast, the present review focusses almost exclusively on vimentin, and covers both ex vivo and in vivo data from tissue culture and from living organisms, including a summary of the many phenotypes of vimentin knockout animals. Our aim is to provide a comprehensive overview of the current understanding of the many diverse aspects of vimentin, from biochemical, mechanical, cellular, systems biology and medical perspectives.

## 1. Vimentin: An Introduction to the Protein

The intermediate filament protein vimentin is expressed in the cells and tissues of many different organisms. The expression of vimentin variants showing high sequence homology and similar expression in major tissues in organisms down to shark, indicates that vimentin has an evolutionary role [1]. Although vimentin was first described in a limited number of physiological and pathophysiological contexts, more recent findings have suggested that it has diverse roles across a broad range of cell and tissue functions and is coupled to a large variety of human diseases. Such diseases include cataracts, cancer, Crohn’s disease and HIV [2,3,4,5]. A number of review articles on intermediate filament proteins have included information on how vimentin regulates important cellular and tissue functions [6,7,8,9,10,11,12,13]. Other reviews have focussed mainly on the in vitro protein properties of the vimentin molecule [14,15,16], and the role of vimentin in specific diseases [3,17,18,19]. In contrast, the present review is designed to provide an overview of the diverse functions of vimentin across a wide variety of physiological and pathophysiological conditions. First, we describe the basis for the high structural plasticity of the vimentin molecule, and the localization and function of vimentin in cells and tissues. The primary focus of this review is an ex vivo (i.e., in cell and tissue cultures) and in vivo (i.e., in living animals) perspective to describe role of vimentin the control of cellular functions and the manifold phenotypes of vimentin knockout animals. Furthermore, we summarize vimentin’s implication in a large set of diverse diseases and the potential of vimentin as a clinical biomarker or drug target. Finally, we speculate about the basis for the functional diversity of this protein and suggest avenues for the focus of future research.

Together with microfilaments and microtubules, the highly insoluble intermediate filaments make up the basic cytoskeleton of metazoan cells. Functional interconnection between these three systems maintain cellular structure and shape, and regulate the biochemical, mechanical and spatial properties of the cell (for an overview, see [6,8,13]). The current biological understanding of intermediate filaments stems from the advances in microscopy techniques in the late 1970s and early 1980s, when research in the field of intermediate filament biology boomed. Intermediate filaments appear to have already been identified in the late 1960s by Buckley and Porter [20]. However, it was not until the 1970s that these were named as such, based on their localisation between filaments of actin and myosin in muscle cells. These early-identified intermediate filaments were, however, not built of vimentin, but of desmin, which at that time was named “skeletin” [21]. An alternative interpretation of the name, that appears to have wide acceptance among researchers in this field, is that it relates more to the intermediate size of these filaments than to their intracellular position. Thus, according to this interpretation, intermediate filaments refers to the diameter of these filaments as intermediate, as compared to the other two major components of the cytoskeleton, actin and microtubules [22,23]. Indeed, as these “intermediate” filaments can be defined for any subcellular position in non-muscle cells, it appears more plausible to associate the term with size rather than position.

### 1.1. Primary and Secondary Structure

The intermediate filament proteins have been classified into six categories based on their sequence homology, as Types I to VI [24]. Here, we consider the Type III intermediate filaments that comprise vimentin, desmin, glial fibrillary acidic protein (GFAP) and peripherin [24,25]. The first Type III class gene to be cloned was that of vimentin; the complete cDNA was first cloned in hamster in 1983 [26,27,28]. Characterisation and cloning of parts of the vimentin gene was also performed in chicken and mouse models in 1983 [29,30]. This was followed by the partial mapping of the human gene in 1985, with the complete sequence published in 1988 [31,32]. Ten transcripts of the human gene (*VIM*; chromosome loci: 10p13) have been identified to date. Of these, only four are protein coding with two translated into the 57 kDa, 466 amino acid, vimentin protein (UniProtKB; P08670 VIME, CCDS 7120.1) [32].

**Figure 1:**
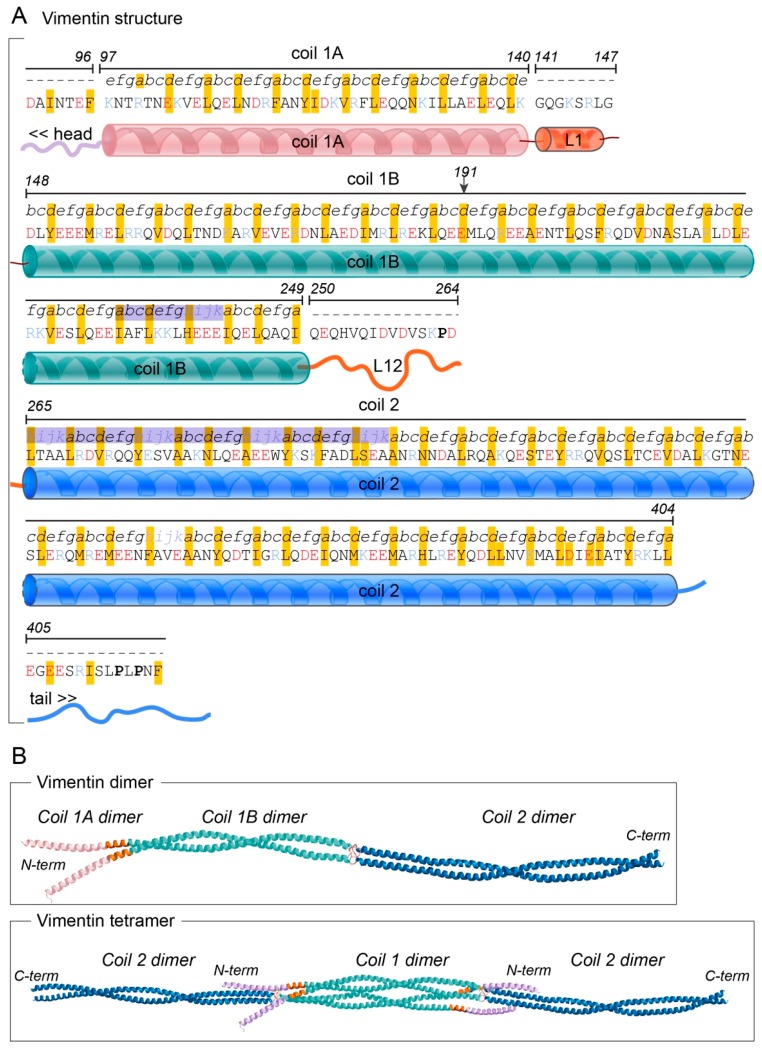
(**A**) The amino acid sequence of vimentin rod domain, with the amino acid position in the sequence indicated above the one-letter code. Heptad (not coloured) and hendecad (violet) motifs are indicated. Residues predicted to be buried in the hydrophobic core are highlighted in yellow. The coil 1A, linker 1, coil 1B, coil 2 structures are indicated with pink, red, violet, and blue, respectively. (**B**) Schematic structure of a vimentin dimer (top) and tetramer (bottom) showing the antiparallel association of two coiled coil dimers, with the structures indicated with colours as in A. Each dimer is formed by a pair of parallel chains. The figure is adapted from Figure 4 in Chernyatina, PNAS 2015 [36], and based on data by Chernyatina, PNAS 2012 [37].

The vimentin protein consists of a central, 310-amino-acid-long α-helical rod domain, which contains 70 acidic and 46 basic amino acids (Figure 1A) [32,33,34]. The N-terminal part of this rod domain is a coil 1 motif, which is organised into sequences of seven amino acid residues (i.e., heptads), where every first and fourth residue is hydrophobic. The C-terminal half of the rod domain has a coil 2 motif that shows a hydrophobic pattern with a different periodicity, 11-residue-long repeats (i.e., hendecads), where the first, fourth and eighth residues create a hydrophobic core (Figure 1A) [35]. In contrast to the acidity of the long α-helical rod, the domain located N-terminally to the rod domain, known as the head domain, has been proposed to have a basic character due to 12 arginine residues in this 102-amino-acid-long sequence [34].

### 1.2. Tertiary Structure

Crystallographic studies and prediction models have revealed that the periodic hydrophobic patterns of the coiled-coil heptad and hendecad segments in the central rod domain result in the formation of relatively regular left-handed coiled-coil dimers [27,36,37,38]. The N-terminal section of the coil 1, defined as coil 1A, shows the predicted heptad pattern, and it is followed by the rigid, α-helical linker L1 structure, which causes a shift in this pattern (Figure 1B). These domains are followed by the C-terminal part of coil 1, defined as the coil 1B domain, which has been predicted to mainly have a regular α-helical structure with a heptad pattern that results in a left-handed coiled-coil structure [37,39] (Figure 1B). The C-terminus of the coil 1B is connected to a short β-strand linker, L12, which can function as a flexible hinge to connect the relatively rigid coil 1B to the adjacent coil 2 domain (Figure 1B) [36].

Both the N-terminal and C-terminal regions of the coil 2 domain contain repeats that are conserved between different intermediate filament proteins. The hendecad repeats of the N-terminal region form parallel α-helical bundles [34], and this process is facilitated in vimentin by the N-terminal capping by the Pro263 residue (Figure 1B). The remaining part of coil 2 has been shown to be a regular left-handed coiled-coil structure that form two intertwined helices, which together form a rigid structure that appear to be important for the mechanical properties of vimentin [34]. Hence, the vimentin protein consists of structurally different domains. The observation that different parts of the vimentin gene show higher degrees of similarity to the corresponding regions in the different Type III genes desmin or GFAP suggests that different domains of the protein might have been subjected to different evolutionary pressures [40]. This indicates that different domains of vimentin might have evolved independently of each other, and therefore would have clearly defined, and distinct functions, which might contribute to the functional diversity of this protein.

### 1.3. Protein Assembly

The assembly of intermediate filaments is fundamentally different to the assembly of actin microfilaments and microtubules. While globular actin and microtubule proteins form polar filaments, the intermediate filament dimers assemble in a stepwise process into non-polar filaments [36]. Furthermore, while the assembly of actin microfilaments and microtubules is dependent upon nucleotide triphosphates, vimentin assembly occurs spontaneously in vitro. However, findings indicate that in an ex vivo context, the initial steps of vimentin assembly are indeed ATP-dependent [41]. Experiments using X-ray crystallography and electron paramagnetic resonance have shown that in vitro, the initial, rapid event in the assembly of vimentin into filaments is the formation of a 46-nm-long dimer by the parallel alignment of the rod 1 domains of two different vimentin polypeptides. The coil 1A and the linker L1 domains are important for the assembly of these dimers (Figure 1B) [37]. In vitro data have further shown that two dimers then can interact in an anti-parallel manner. The two glutamic acid residues at position 191 of each sequence are in close proximity within the interphase between the protein dimers, which associate laterally into a non-polar tetramer (Figure 1B) [35,37]. This event appears to be promoted by the complementary charges on the different dimers [37]. In particular the lysine residue of coil 1A at position 139 and the positively charged linker L1 of one dimer appear to interact with the negatively charged C-terminus of coil 1B of the other dimer [34,37,42]. Furthermore, the head domain of vimentin has an essential role in the tetramer formation, due to attraction between the 12 basic arginine residues of the head domain and the charges of the acidic rod domain of the second dimer [34,39]. Thereafter, eight tetramers assemble laterally to make up the unit-length filament (ULF), which consists of a central core of 32 coil 1 domains and two flanking segments of 16 coil 2 domains [43]. However, in vitro, vimentin filaments can also assemble with as few as four or as many as 12 tetramers per cross section [16,44]. In the slower elongation step, these individual ULFs anneal in a longitudinal manner via the coil 2 domains, which results in the molecular rearrangements within the individual ULFs. The formation of the short filament units consists of two, three, or more ULFs [39,43]. During this last phase of assembly, the filaments undergo a radial compaction to form long compacted filaments of vimentin of a diameter of ~10 nm [43,44,45,46,47,48]. Hence, the vimentin intermediate filaments are built from coiled-coil multi-domain amino acid sequences that are stabilised and connected by hydrophobic and ionic interactions, to form the fibrous proteins [34,35,46,49,50,51,52,53].

The vimentin structure is dynamic in cells and can be rearranged to form filaments or the soluble tetrameric protein, ULF particles [45,46,51,53]. Studies using live-cell imaging have shown that vimentin filaments undergo constant and relatively rapid changes to their shape and localisation. However, in contrast to actin and microtubule polymerisation, vimentin filaments do not appear to completely disassemble, reassemble and exchange units with the soluble pool. Rather, vimentin filaments remodel by constant severing and reannealing of small clusters ex vivo, which allows for turnover of vimentin while maintaining the length and structural integrity of the network [43,46]. As mentioned above, the available evidence indicates that vimentin filaments can assemble with various amounts of tetramers per cross section, and filaments with more than the standard eight tetramers per cross section have been proposed to form subunits that are more loosely bound and therefore predisposed to release vimentin subunits [7,54]. The ULFs and the soluble pool of vimentin tetramers have been observed to interchange molecules ex vivo [55,56], which can result in additional oligomeric forms of vimentin. It is important to note that, according to Wickert et al., 2005, vimentin can also assemble with other Type III intermediate filament proteins, to form heterodimers that assemble into heterodimeric polymers [57]. As a consequence, the vimentin molecule is believed to be involved with diverse types of multiprotein complexes, which will provide great biochemical and functional diversity.

### 1.4. Post-Translational Modifications

Vimentin is also known to undergo several post-translational modifications [58,59] that can regulate the functional properties of vimentin in the context of health and disease [59,60]. Concomitantly, crosstalk between different types of post-translational modifications extends the possibilities for the regulation of vimentin function. The range of post-translational modifications that have been reported to modify the vimentin protein includes phosphorylation [60], sumoylation [61] acetylation [62], glycosylation [63], glycation [64] and ubiquitination [65]. Most of these post-translational modifications have been shown to regulate the solubility of vimentin in vitro, e.g., sumoylation [59,61]. Recently, glycosylation was found to be required for the assembly of vimentin filaments in cells [66]. The head and tail domains of vimentin contain more than 35 phosphorylation sites [7] (PhosphoNET database). Current ex vivo data suggest that phosphorylation of vimentin serine residues inhibits its subunit polymerisation, thus promoting the disassembly of vimentin filaments and increasing the solubility of the protein [8,67,68,69]. Phosphorylation of vimentin has also been shown to regulate protein‒protein interactions and intracellular signalling [59,70]. The observation that hyperphosphorylation of intermediate filaments can be linked to numerous diseases including cancer, has indicated that the phosphorylation status of vimentin can be important for health and diseases [59,71].

Taken together, these reports show that biochemical diversity of vimentin can be promoted by the various sizes of the molecule, its assembly state, its potential to co-assemble with other types of intermediate filaments and its posttranslational modifications. Additional factors can control the properties of vimentin such as interactions with non-intermediate filament proteins. For example, molecular chaperone activities towards vimentin have been reported to inhibit filament polymerisation [72] and mutations in chaperones reduce their ability to interact with vimentin and lead to vimentin aggregation [73]. In addition, Vmac, a protein in rat kidneys, has been reported to regulate cellular morphology by binding, and regulating the dynamics and organisation of vimentin filaments [74]. Given recent data showing that approximately 1% of the entire human proteome (N = 186) localises to the intermediate filament network within cells [75], there might be a large number of unidentified vimentin-associated and vimentin-regulating proteins.

### 1.5. Vimentin: Location and Timing

**Figure 2:**
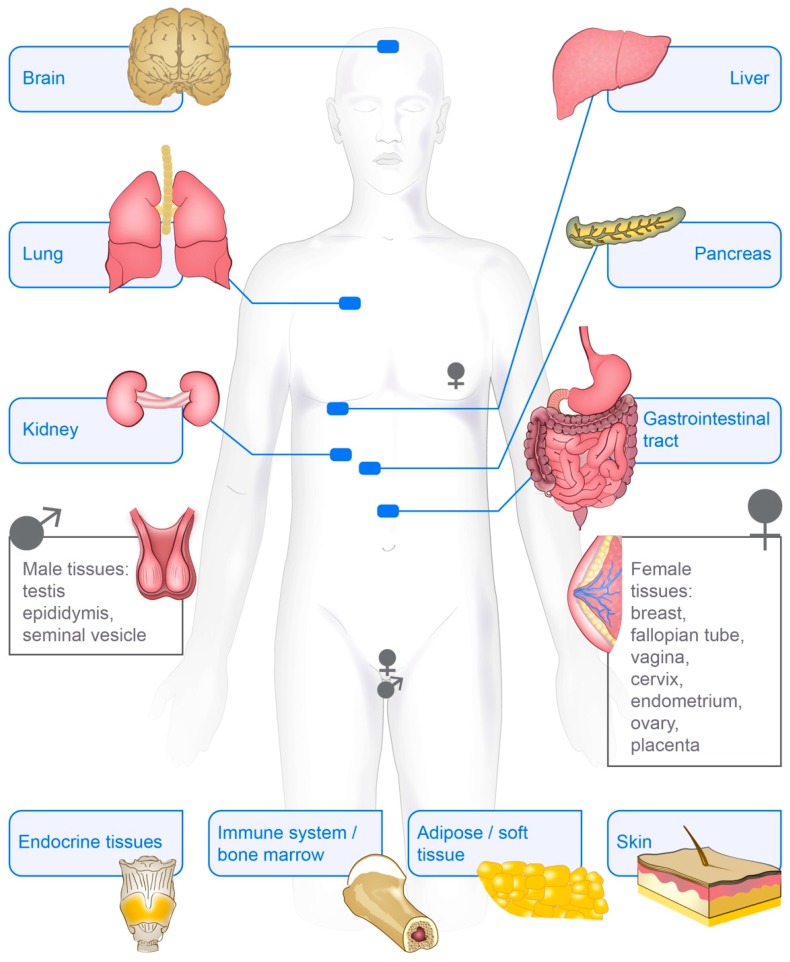
Example of tissue groups in which vimentin protein has been identified in low, medium or high levels. Gender-neutral and gender-specific tissue groups are indicated by blue or white squares, respectively.

The early studies of vimentin showed that the protein was present in bovine eye lenses [76,77]. Further studies in chicken and mice showed expression of the vimentin gene mainly in cells of the connective tissue and central nervous system as well as in erythroid and muscle cells [30,78]. In studies of the development of muscle and neural tissues in chickens and hamsters, vimentin mRNA was further found to mainly be expressed in highly proliferative and undifferentiated cells [79,80]. In humans, later expression studies reported the vimentin gene to be constitutively expressed across all major tissues, as shown in the three major databases for RNA expression: HPA, GTEx and Fantom [81,82,83]. In the Human Protein Atlas database, the vimentin protein was found to be expressed in the majority of the 44 tissues analysed, 14 of which showed high levels of vimentin expression. These tissues included skin, lung, kidney, bone marrow and lymph node (Figure 2), https://www.proteinatlas.org/ENSG00000026025-VIM/tissue [81].

In cells, vimentin forms a network that surrounds the nucleus. From there, it extends throughout the entire cytoplasm, with shorter soluble forms more abundant in the cell periphery [8,23,84]. Efficient transport of vimentin is required for maintenance of the intracellular network. Vimentin transport has been shown to occur along microtubules, and to depend upon actin filaments, microtubules, the microtubule-associated motor protein kinesin-1 and the cytoskeletal regulators PAK and ROCK [84,85,86]. The filaments can extend to the plasma membrane and are able to attach to the nucleus. The soluble shorter forms of vimentin are found inside the cell, on the cell surface and in the extracellular environment. The organisation of the filamentous intracellular network of vimentin varies in different cells. For example, it can form relatively homogenous distributions within the cytoplasm of primary, senescent or non-dividing mesenchymal cells, and is rapidly reorganised towards the nucleus upon exposure to PDGF, oncogenes or viruses [87,88]. The subcellular spatial organisation of vimentin fibres is regulated by the post-translational modifications and by the assembly state and solubility of vimentin [41,89,90]. The many different forms of vimentin can result in binding of different types of associated proteins and protein complexes.

## 2. Vimentin: Function

### 2.1. Knock-Out Mouse

When the mouse gene knockout techniques became available in the 1980s, mice that lacked genes encoding actin, tubulin and vimentin were created. In contrast to the actin and tubulin knockout mice that died in early embryogenesis, the first study of vimentin knockout mice showed that the mice developed and reproduced with no obvious defects [91]. Vimentin was therefore called “The conundrum of the intermediate filaments” [92]. However, later studies observed many, diverse defects in vimentin-null mice.

To summarise these findings, we searched for vimentin knockout mouse phenotypes in the bioinformatics resource database, Mouse Genome Informatics, and in the published literature. More than 30 different phenotypes have been reported for vimentin-deficient mice, as summarised in Table 1. The most commonly reported defects reported in vimentin knockout mice are the loss of cell morphology and reduction in cell adhesion, polarisation, stiffness and migration. At the organ level, these defective cellular functions have been linked to reduced wound-healing capacity, and inability to properly remodel arteries and vasoactivity. At the organismal level, vimentin knockout was connected to cataracts, hyperactivity, impaired balance, impaired coordination, and increased anxiety-related responses. In addition, loss of vimentin has been reported to reduce inflammation and infection (for references, see Table 1). These in-vivo mouse knockout phenotypes indicate the functional role of vimentin in physiological systems, and can bridge the gap between in vitro, ex vivo and clinical data. Although most of these studies are in line with a role for vimentin in the control of cell shape, architecture, stiffness, adhesion and migration, they also point to roles of vimentin in signalling and in many aspects of human diseases as will be described further below.

### 2.2. The Role of Vimentin in Cytoskeletal Cross-Linking and Intracellular Organization

The three different cytoskeletal filament systems of the cell are interconnected by protein-protein interactions, and changes in one of these filamentous systems often results in changes in the other systems [110,111]. This cross-talk provides flexibility to the cytoskeleton as a whole and allows it to be constantly rearranged to meet the needs of the cell under various conditions, such as cell migration and through the course of the cell cycle. Since the early 1980s, it has been widely accepted that vimentin and microtubules are connected to each other, and that this connection is important for the localisation and function of vimentin (for overview, see [112]). More recently, it was found that the tumour suppressor, adenomatous polyposis coli (APC), can function as a bridge between these cytoskeletal filaments [113]. Small units of vimentin have been observed to be transported by microtubule-dependent motor proteins towards the cell periphery, where they can join and form longer filaments that are later incorporated into the cellular network [84,85,86,114,115,116,117,118]. However, it is important to note that the transport of vimentin is bidirectional and governed both by microtubules and microfilaments [85,86].

Actin and vimentin have been observed to localise to the same subcellular environment. For example, vimentin localises to the microdomain prior to podosome formation [119]. The tail domain of vimentin has been reported to interact with actin both directly [120] and via cross-linking proteins such as plectin [121]. As a consequence, versal F-actin stress fibres and transverse arcs interact with vimentin, and the retrograde flow of these actin filaments results in retrograde transport of vimentin filaments from the cell front [122]. Reduced levels of vimentin have also been shown to result in increased stress fibre assembly and contractility, and, notably, this effect was reversed upon expression of filamentous vimentin but not vimentin ULFs [123].

Vimentin-null mouse embryonic fibroblasts show increased motility of organelles compared to wild-type fibroblasts [124,125]. Together with the observations that vimentin can interact with Golgi, and control the localisation of mitochondria, the late endosomal‒lysosomal compartment, and the nuclear size and shape [126,127,128,129,130], this indicates that vimentin might also regulate and stabilise the organisation of organelles.

Vimentin might also have a more general role in protein function in the cytoplasm. It has long been known that inclusion bodies of misfolded proteins are surrounded by a ‘cage’ of vimentin [131]. Recent data have shown that this cage is formed prior to the accumulation of misfolded proteins, which suggests that vimentin might have a general role in protein quality control in the cytoplasm [132]. Vimentin, unlike actin and microtubules, is not divided symmetrically between two daughter cells during mitosis, rather it is localised mainly to one daughter cell [132]. Taken together, these observations suggest that vimentin has a role in the organisation of, and protection against misfolded proteins in cells.

### 2.3. Cell Mechanics

Cell mechanics relate to cellular parameters such as the elastic or viscous responses of cells after application of external forces (for overview, see [133]). An understanding of these mechanical properties is necessary to define how cells interact and respond towards environmental changes, such as during cell invasion and migration. Mechanical properties are adaptable and can be altered, e.g., in pathogenic cases such as cancer [134]. The mechanical behaviour of cells is mainly determined by the nucleus and cytoskeleton, including cytoskeletal crosslinkers and molecular motors [135]. Over the years, actin and microtubules have been extensively studied in order to determine their particular influence on the mechanical behaviour of cells (for overview, see [135]). In contrast, the effects of intermediate filaments on cell mechanics has only been recently investigated, even though the mechanical properties of intermediate filaments in vitro have been studied for more than two decades [133].

The mechanical responses of cells to applied forces depend on several parameters influencing the mechanical results obtained. It is possible to classify the observations of vimentin-dependent control of cell mechanics based on the following methodological parameters: time scale of deformation (i.e., fast, slow, short, long) [136]; repetitions of measurements, such as creep, step and oscillatory (sines, cosines) measurements; pushing together or pulling apart of cells [137] or the way of interfering with vimentin (e.g., chemicals [withaferin A, acrylamide], oncogenes, cytoskeletal crosslinkers (e.g., plectin), vimentin knockout cells, RNA, interference). Further parameters include the amount of strain (i.e., small versus large); if the analysis is performed in vitro, ex vivo, or in vivo; if elastic or viscous properties are probed [138]; if cells are deformed globally as whole cells or rather locally on a subcellular level; and if cells adhere to a substrate during measurements or are in suspension. Finally, many of these parameters of measure depend on the tool used for measurement. We could show previously that state of adhesion (e.g., adhesive cells versus same cells in suspension) governs the mechanical response [138]. Recently, it was shown that the direction from which cell mechanics is probed can also have effects on the mechanical responses, e.g., smaller or larger values are added to the nominal values, depending on the geometric setting [139]. Therefore, the manner in which the forces are applied is important, such as in the direction of or orthogonal to the polarisation of the cells, or in a rotational manner. It is also clear that mechanical responses depend upon whether a cell is probed locally or globally. Here, the available data on the role of vimentin in cell mechanics are therefore classified according to the methodological approaches used to acquire the data, with particular focus on the state of adhesion while the cells are probed (as described in Table 2).

The many and different types of probing methods used in studies of vimentin and cell mechanics have shown that it is difficult to obtain a clear picture from the studies in the literature. The same probing techniques have been used with different cell types, and different methods have been used to alter vimentin levels, with miscellaneous data generated regarding the role of vimentin in cell mechanics (see Table 2). This indicates that the method used to alter vimentin can influence the response, as described earlier by Charrier and Janmey [150]. Although a number of studies have indicated that vimentin is important for generation of contractile forces in cells [143,144], a recent study reported that the loss of vimentin increased the generation of contractile stress 3-fold [146]. Moreover, the viscous responses of cells have been suggested to depend upon vimentin [151]. In particular, many lines of research indicate that the amount of vimentin correlates with the stiffness of cells (Table 2). It is important to note that most of these studies have been based on probing methods that squeeze adherent cells. A comprehensive, comparative study using the same method to interfere with vimentin in adherent and suspended cells, and that probes cellular mechanics, as both pulling and pushing the cell would be of importance for studies in the future. Such a study would reveal the full impact that vimentin might have on the mechanical properties of living cells.

### 2.4. Cell Adhesion

#### 2.4.1. Focal Adhesions

Cell‒matrix adhesion, focal contacts and focal adhesions are large protein assemblies that connect the cell to the extracellular matrix via integrins, and that mediate biochemical and mechanical signals from the cell surroundings. Vimentin has been shown to be spatially localised to cell‒matrix adhesions in different cell types. While small squiggles or particle units of vimentin have been observed to localise to small nascent adhesions, filamentous vimentin has been associated with mature, large focal adhesions [152]. Long focal-adhesion-associated filaments have been observed at points where F-actin stress fibres reach the focal-adhesion area [119]. Vimentin can bind directly to the cytoplasmic tail of integrin α2β1 and it is enriched in integrin-β1-containing focal adhesions [153]. The spatial localisation of vimentin at focal adhesions depends upon integrins [154]. The intermediate filament system associates with both integrin α6β4 [155] and plectin [156]; vimentin has been shown to bind to focal adhesions and fibrillar adhesions via the plectin isoform 1f (P1f) [157]. The binding of vimentin and P1f has been shown to result in capture and fusion of mobile vimentin precursors, to form a de-novo intermediate filament network close to the focal adhesions [157]. This interaction has been shown to promote the lifetime, stability and mature morphology of focal adhesions. Kim et al. (2010) showed that vimentin directly interacts with filamin A to stabilise cell adhesion [158], and that this filamin A–vimentin interaction is necessary for cell adhesion to collagen [159]. Interactions between vimentin and the actin cross-linking protein fimbrin might also control cell adhesion [160]. Additionally, vimentin has been shown to promote factors that can enhance integrin activation, such as the clustering and ligand affinity for β3 integrins [161] and ligand affinity of β1 integrins [162]. Taken together, these studies suggest that vimentin promotes integrin clustering and activation, and thereby controls the organisation and dynamics of focal adhesions. This concept is supported by a number of additional observation, e.g., vimentin stabilises cell‒matrix adhesions that are subjected to shear stress [163]; the focal contacts of fibroblasts from vimentin-null mouse cells are more irregular, less distinct and less mechanically stable, compared to wild-type control cells [140]; and thick vimentin bundles have been associated with large focal contacts, while decreased vimentin levels result in reduced sizes of focal contacts [163]. In addition to these observations that support a role for vimentin in promoting focal adhesion growth, recent findings showing that cells that lack either plectin or vimentin show larger focal adhesions, indicate that vimentin also can restrict the size of focal adhesion. However, upon loss of tension, the enlarged focal adhesion phenotype was reduced in both cell types, indicating that cytoskeletal tension is required for vimentin or plectin to limit the size of focal adhesions [164]. A role for vimentin in focal adhesion dynamics is further indicated by the observation that expression of vimentin in epithelial cells increase turnover of the protein paxillin within focal adhesions by 4-fold [165].

The induction of lamellipodia and the concomitant disassembly of focal adhesions, result in filamentous vimentin to be broken down into smaller units [166]. A comparison of the nanoscale structures of vimentin and adhesions in normal and oncogenically modified fibroblasts showed that the increased homogenous distribution and increased density of nanoscale adhesions in the modified cells were linked to loss of directionality and increased entanglement of vimentin filaments [167]. Together, these observations indicate that vimentin controls the organisation, structure and function of cell‒matrix adhesions, possibly through long vimentin filaments, which result in strong, well-organised, distinct, mature, stable and mechanically resistant focal adhesions, while short forms of vimentin might have opposite effects on the stability of these adhesions.

#### 2.4.2. The CD44 Receptor

Hyaluronan (hyaluronic acid) is a glycosaminoglycan that is widely found in epithelial, connective and neural tissues. It is synthesised at the inner side of the plasma membrane and exported to the extracellular environment by transmembrane transporter proteins [168]. It is a major component of the extracellular matrix. Its primary receptors are CD44 and RHAMM, which both function in cell adhesion. The CD44 receptor is crucial for adhesion in a hyaluronan-based cellular matrix [169,170,171,172,173]. Vimentin has also been reported to provide a direct binding site for CD44 on its N-terminal head domain [173], and upregulation of hyaluronan has been shown to be correlated with increased vimentin levels [174]. Taken together, these data suggest that vimentin–hyaluronan interactions mediated by the CD44 receptor might have roles in cell adhesion.

#### 2.4.3. Extracellular Vimentin

So far, the role of vimentin within the cell has been considered. However, vimentin is also present on the surface of cells, as well as in the extracellular matrix, e.g., via secretion by activated macrophages [175,176] or astrocytes [177,178]. Experiments using Golgi blockers have shown that extracellular vimentin is actively secreted through the Golgi apparatus [175]. This extracellular vimentin can bind to specific cell-surface receptors to activate them, e.g., by phosphorylation, as is the case for insulin-like growth factor 1 [179]. Such an approach has been used experimentally to enhance axonal growth both ex vivo and in vivo, with significant improvements in the recovery of spinal cord injured mice [180,181]. In another case, extracellular vimentin was shown to bind to the anti-LOB7 antibody, which resulted in significant increase in the formation of tubes of endothelial cells after 5 h [182]. Additionally, extracellular vimentin has been shown to have a role in inflamed tissue in atherosclerosis [183] and to interact with von Willebrand factor in platelets [184]. Specifically, vimentin reduces the adhesion of human leucocytes to platelets and the endothelium by binding to P-selectin on leukocytes and endothelial cells, which resulted in the suggestion to use recombinant vimentin to attenuate inflammation [185].

An alteration to secreted extracellular vimentin can indicate a pathology. For example, the citrullinated form of vimentin is expressed in patients with rheumatoid arthritis, as indicated further below (3.11.). Detection of the citrullinated form of vimentin is thus used as an early marker for this autoimmune disease [186,187,188]. These observation are in line with the finding that mice lacking vimentin show increased production of reactive oxygen species, which suggests that vimentin influences the immune response and supports inflammation through reduction of the bactericidal capacity of macrophages [103]. Vimentin has also been reported to be present on the cell surface, as physically attached to the cell membrane. In this location it has been shown to interact with CD44 in umbilical vein endothelial cells [173], to bind to and inhibit the internalisation of human papillomavirus [189] and to interact with listeria [190,191]. Surface vimentin has also been suggested to promote infection by binding to dengue virus [192]. These observations suggest that extracellular vimentin might modulate the functions of cell-surface receptors. Vimentin has also been observed in the exosomes and microparticles in body fluids from different disease conditions [193,194], suggesting that also vimentin in extracellular small vesicles might serve as clinical markers for diseases.

### 2.5. Cell Motility

Cell motility is required for various types of physiological phenomena, such as tissue repair, homeostasis and wound healing. A large variety of different types of cell motility have been described, such as mesenchymal, amoeboid, pseudopodial and lobopodial migration [195,196,197,198,199]. Regardless of the mode, migration requires acto-myosin contractile forces, interactions with the extracellular environment, and the capacity to move the cell body forwards [200]. Vimentin has been reported to regulate all of these factors [199]. For example, vimentin-deficient mice show defects in motility and directional migration of fibroblast, as well as reduced capacity to heal wounds [105,201,202]. Close correlation has been observed between the local disassembly of the vimentin network in migrating cells and the formation of a lamellipodium, used for pseudopodial migration [166]. The width of the lamellipodium has been suggested to be influenced by vimentin, which will restrict the actin retrograde flow in migrating cells [122]. In addition, vimentin-deficient lymphocytes show defective adhesion and migration [97] and cleavage of the vimentin is important for cell invasion [11]. Furthermore, increased soluble fraction of vimentin and loss of directionality of vimentin fibres are linked to increased cell invasion ex vivo [88,167]. These results suggest that vimentin is a regulator of cell migration [202], although the exact mechanisms by which it interacts with the force-producing actin–myosin machinery have not been identified yet.

### 2.6. Epithelial‒Mesenchymal Transition

During epithelial‒mesenchymal transition (EMT) epithelial cells undergo functional and behavioural changes to differentiate into mesenchymal cells [203,204]. This event is crucial for embryonic development, tissue regeneration, homeostasis and wound healing, and is also necessary for formation of metastases in cancers. These changes are marked by key events, including loss of epithelial cell‒cell junctions, switch from apical‒basal polarity to front‒rear polarity, morphological changes of cell shape, and reorganisation of the cellular cytoskeleton (for review see [205]). Upregulation of vimentin is a canonical marker for EMT [206], as mesenchymal cells are vimentin dominated [207], and the expression of vimentin promotes the transformation of cells to a flatter, elongated, mesenchymal shape [165]. Furthermore, vimentin interacts with microtubules and associated motor proteins [116,208,209,210], which can promote cell motility. Phosphorylation of vimentin also sequesters members of the 14-3-3σ family [211], which are important factors in EMT due to their control of Akt phosphorylation [212]. An active form of Akt phosphorylates vimentin at Ser39 and upregulates vimentin to promote further motile and invasive behaviour [213].

### 2.7. Invadopodia, Filopodia, Lamellipodia and Microtentacles

Invadopodia are actin-rich, finger-like projections that can breach the plasma membrane of invasive cells and degrade the extracellular matrix. Actin polymerisation is the driving force for elongation of invadopodia, which arise from lamellipodia and filopodia [214,215,216,217,218,219,220,221,222]. Lamellipodia are flat membrane protrusions that contain a meshwork of actin bundles [223]. Filopodia are parallel actin structures that extend beyond lamellipodia, as the leading edge of invadopodia [224,225,226]. While invadopodia formation is reliant on actin, vimentin is required for the elongation of invadopodia [227,228]. Vimentin further increases the invasive properties of cells via cGMP-dependent kinase phosphorylation of VASP [229]. An additional motile property of vimentin is seen within the leading edge of lung cancer cells, where vimentin can bind to and regulate focal adhesion kinase (FAK) [230]. Vimentin can regulate cell adhesion by recruiting the active variant of the Rac1 GEF VAV2 to focal adhesions [230]. As cellular adhesion is correlated with motility, upregulation of vimentin is expected to increase the motile behaviour of cells.

Serum stimulates phosphorylation of vimentin, which results in organisational changes to vimentin filaments, and formation of lamellipodia [166]. This event might be important during EMT, where vimentin expression drives the transition to mesenchymal cell shape, accompanied by enhanced motility and focal adhesion dynamics, again pointing to interconnections between vimentin and other cytoskeletal systems within cells.

Microtentacles are another type of membrane extension that are microtubule based, rather than actin based. These membrane extensions have been shown to help circulating tumour cells to reattach at the vasculature when leaving the blood stream, on their way to metastasis formation [231]. Microtentacles have been shown to contain vimentin [232,233], although the definition of the role of vimentin in these protrusions requires further studies.

### 2.8. Cholesterol Metabolism

A number of studies have suggested a link between vimentin and lipids. For example, cell lines that lack vimentin show notably lower storage of LDL-derived cholesterol than control cells [234], and reduced transport of LDL-derived cholesterol from lysosomes to the esterification site [235]. Immunofluorescence-based microscopy studies have shown that vimentin is closely associated with lipid droplets in cells [236].

### 2.9. Vimentin Control of Cell Proliferation, Apoptosis and Differentiation

A number of observations have indicated that vimentin controls cell proliferation. For example, factors that promote cell proliferation, such as platelet-derived growth factor, serum (but not platelet-poor plasma), and insulin or epidermal growth factor, have been reported to increase the cytoplasmic vimentin mRNA levels in mouse 3T3 cells [237]. Loss of vimentin has been shown to reduce the proliferation of fibroblasts, and reduce the levels of a major initiator of EMT, TGF-β1, a phenotype that can be reversed upon re-expression of vimentin [104]. Expression of oncogenes that result in increased proliferation of cells has been reported to result in higher levels of vimentin mRNA and vimentin protein levels, and in increased fractions of the soluble form of vimentin [88]. Also, cell apoptosis has been reported to depend upon vimentin, and more specifically for proteolysis of vimentin into a pro-apoptotic amino-terminal fragment of vimentin that can induce apoptosis [238].

With regard to differentiation, in various model systems, the levels of mRNA and the vimentin protein have been shown to be inversely proportional to differentiation of muscle, lens, bone and neuronal tissue cells [239,240,241]. Furthermore, the lack of vimentin blocks the EMT-like transdifferentiation of keratinocytes, which occurs during wound healing. This link between vimentin, cell proliferation and suppressed differentiation has previously been highlighted by Hol and Capetanaki 2017 [242], and that vimentin expression in differentiating cells might be replaced by a tissue-specific intermediate filament protein in the fully differentiated cell. The observation that decreased phosphorylation of vimentin results in increased neuronal differentiation for ex vivo neuronal progenitor cells [243] suggests that vimentin phosphorylation is important for vimentin-dependent control of cell differentiation.

Hence, it is possible that vimentin promotes cell plasticity, as the capacity of cells to change, either by forming new cells through proliferation, or by differentiating into new types of cells. Stem cells can give rise to multiple cell lineages and transdifferentiate into different cell types in their respective environments. Indeed, the role of vimentin in the plasticity of stem cells is an area for future research.

### 2.10. Vimentin-Dependent Control of Protein Signal Transduction and Gene Transcription Involved

Intermediate filament proteins do not only serve to maintain the structural integrity of the cell, they also have important roles in various intracellular signalling systems, such as TGF-β1 EMT [104], Slug- and Ras-EMT [244], 14-3-3-, extracellular signal–regulated kinase (Erk)-, and pathway-AKT dependent signalling [18]. A recent review has summarised how vimentin controls biochemical signalling in cells in various physiological and pathophysiological contexts [9]. An emerging theme is that vimentin might act as a scaffold to stimulate signal transduction in cells.

Some observations support a role for vimentin as a regulator of gene transcription. First, vimentin has been reported to bind directly to transcription factors, and thereby suppress osteoblast differentiation [245]. Furthermore, observations of the structural similarities between vimentin and the proto-oncogenes c-fos and c-jun, and oncogenic Raf and the v-mos oncogene have indicated that vimentin-dependent control of gene expression can promote transformation of primary cells into malignant cancer cells [242]. Additionally, viruses, growth factors such as PDGF, and oncogenes have been shown to promote vimentin network relocalisation to the perinuclear ‘cage’ structure [87,88,246]. The observations that vimentin responds to and spatially reorganises upon exposure to various factors that change cellular functions, support the concept that vimentin serves to protect cells against sudden changes.

**Figure 3:**
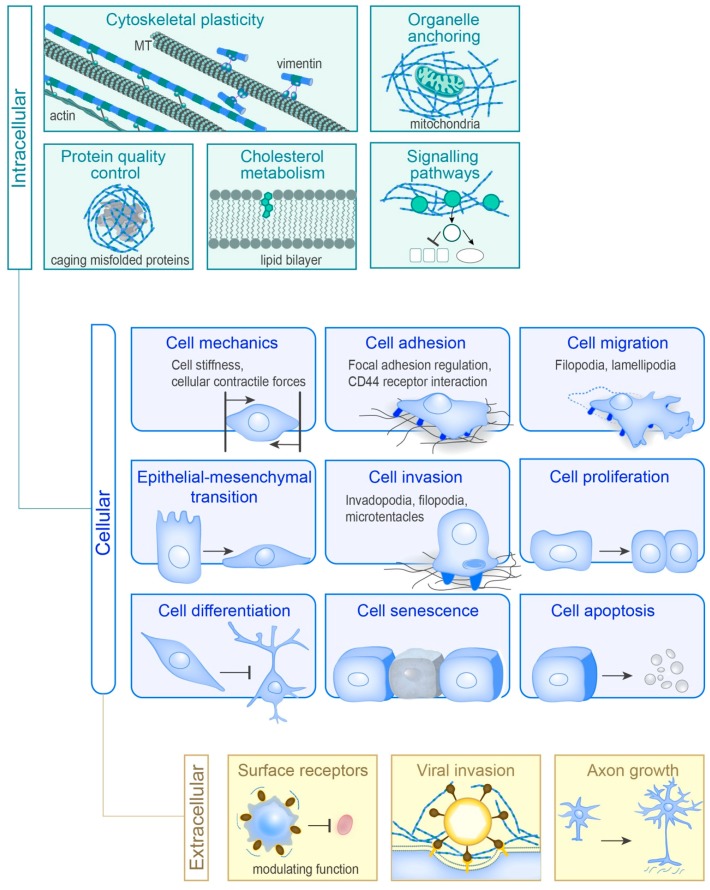
Examples of how the vimentin protein control cells, on a molecular (**top**), cellular (**middle**) and extracellular (**bottom**) level.

Collectively, the information above indicates that vimentin controls several, diverse cellular functions, as summarised in Figure 3. We suggest that vimentin can have many roles in protecting cells under physiological conditions when they are exposed to mechanical forces and biochemical stress linked to tissue remodelling, or even the stress of misfolded proteins. Thereby, vimentin appears to have a general role in facilitating changes, by protecting cells against stress.

## 3. Vimentin: A Drug Target and Biomarker in the Clinic

Vimentin has been linked to a large number of pathological conditions, such as neoplasms, eye diseases, endocrine system diseases, fibrosis-related diseases, heart or vascular diseases, reproductive system diseases, infectious diseases, skin diseases, skeletal system diseases, diabetes, and inflammatory bowel diseases. Below, we briefly summarise the major findings in the studies that have investigated the role of vimentin in disease, across a selection of the best-studied diseases.

### 3.1. Vimentin in Cancer

A variety of processes that occur in cells undergoing malignant transformation and metastasis involve vimentin (for review, see Chen, Eriksson 2016) [18,202]. It is important to note that overexpression of vimentin has been used for decades as a clinical marker for EMT, a critical process in tumour cell dissemination. The initial observations that the expression of the vimentin gene was mainly observed in proliferative and undifferentiated cells were followed by studies that together indicate that vimentin is linked to the malignant transformation and metastatic spread of cancer cells [206,247,248,249].

### 3.2. Lung Cancer

In lung cancer, vimentin expression has been seen in large-cell pulmonary carcinomas, and well-differentiated pulmonary adenocarcinomas. Several studies have provided evidence for the involvement of vimentin in the progression of various lung cancers [18,249]. Its expression has been associated with metastases in non–small-cell lung carcinomas, and lack of differentiation in these cancers [250]. Furthermore, vimentin overexpression in non–small-cell lung cancer is an independent prognostic factor for poor survival [251]. One study of patients with non–small-cell lung cancer showed that expression of vimentin and E-cadherin correlated with favourable patient outcome for erlotinib treatment, which suggested that vimentin has a role as a predictive biomarker for this therapy [252]. In support of this, in lung adenocarcinomas, glycosylated vimentin was shown to be frequently downregulated, defining its potential as a biomarker both for treatment and diagnosis [253]. A more recent study showed that vimentin expression in cancer-associated fibroblasts is required for dissemination of early stage lung adenocarcinoma, which indicates that vimentin might have a key role in the cancer-promoting capacity of cancer-associated fibroblasts in the tumour microenvironment [254]. Fibroblasts that promote the growth of cancer cells in vivo have also been shown to have reorganised vimentin filaments, compared to normal fibroblasts [255]. For further information on the role of vimentin in lung cancer, we refer the reader to the review by Kidd et al. [18].

### 3.3. Breast Cancer

Breast cancer has also been reported to be related to overexpression of vimentin, with correlation with increased invasive behaviour [256,257] and promotion of migration of mammary epithelial cells [207]. Vimentin-associated migration in pre-malignant breast cancer cells has been shown to be induced by H-Ras-V12G and Slug. Interestingly, the presence of vimentin during EMT leads to upregulation of the receptor tyrosine kinase Axl that enhances the migratory behaviour of breast epithelial cells [244]. Korsching et al. (2005) used immunohistochemistry for vimentin and 15 other differentiation markers invasive breast cancer tissue samples, and concluded that neither EMT nor myoepithelial histogenesis could fully explain the origin of the vimentin-expressing cells in the tissues [258].

### 3.4. Malignant Melanoma

Many studies have identified vimentin as a key component of cell invasion and metastasis in malignant melanoma [259,260,261]. Following their proteomic study on malignant melanoma, Li et al. (2010) indicated increased vimentin as a haematogenous indicator of metastasis [262]. In this sense, vimentin might act as a clinical predictor for melanoma, to provide means to predict high-risk patients for haematogenous metastasis, and thus to provide individualised treatment options [262].

### 3.5. Prostate Cancer

Studies have shown an inverse relationship between malignancy in prostate cancer and formation of pancreatic exocrine cells [263,264]. In an in vivo analysis of prostate epithelial cells, vimentin was shown to be key in maintaining homeostasis of the acinus in the prostate. This suggested that vimentin expression in prostate cancer results in high tumorigenic activity. The study showed that vimentin is required for the movement of integrin β1 to the leading edge of the cell, a migratory condition that is necessary for metastasis. Thus, they proposed that a block of the vimentin–integrin relationship represents a potential therapy for metastatic tumours [265]. Another possible treatment targets PKCε, as cell motility is promoted by phosphorylation of vimentin by PKCε [266], and genetic deletion of PKCε has been shown to inhibit development of prostate cancer in mouse model [267].

### 3.6. Gastrointestinal Cancer

In tumours of the gastrointestinal tract, overexpression of vimentin has generally been associated with an increased aggressiveness [268]. Vimentin has been defined for both primary and metastatic tumours of patients with oesophageal squamous-cell carcinoma [269]. In advanced colorectal cancers, vimentin is known to be frequently methylated, which is also used as a diagnostic tool here [270]. A highly sensitive stool test uses vimentin as a biomarker [271,272]. Also, in colorectal cancer, expression of vimentin has been correlated to the stage of neoplastic progression of neoplastic cells. Furthermore, histone deacetylase inhibitor (HDACi)-resistant colorectal cancer cells show overexpression of vimentin compared to HDACi-sensitive colorectal cancer cells. Taken together, Lazarova et al. (2016) suggest that vimentin expression contribute both to the malignancy and drug-resistance of colorectal cancer [19]. Gastric cancer is also associated with vimentin overexpression, and as vimentin is correlated to metastasis formation, it has been suggested to be an indicator of prognosis [273]. Vimentin might also contribute to an invasive phenotype in gastric cancer, which means that it is potentially useful as a biomarker to define cancer aggressiveness [274].

### 3.7. Additional Types of Cancer

Additional cancers witness vimentin overexpression [3] including certain types of lymphomas [275], endometrial carcinomas [276], papillary thyroid carcinomas [277], cervical cancers [278], clear-cell renal-cell carcinomas [279], and central nervous system tumours [280,281]. In summary, the vimentin expression in various types of cancer cells and in cancer-associated fibroblasts appears to be associated with malignancy and drug resistance.

### 3.8. Vimentin in Other Human Diseases

In addition to cancer, vimentin expression has been associated with a number of other diseases. Cellular EMT has a role in many pathogenic pathways, which makes vimentin a key target for many human diseases. Therefore, the use of intermediate filaments as a biomarker for disease is a promising avenue for treatment. Specific mutations in vimentin are correlated with diseases such as cataracts, Crohn’s disease, rheumatoid arthritis, and human immunodeficiency virus.

### 3.9. Cataracts

Cataracts are a common disease that is characterised by the clouding of the lens of the eye. Vimentin filaments have been shown to have an integral role in maintenance of the structure and integrity of the lens [282]. Low levels of vimentin are normally found in the epithelium of the lens, patients with cataracts, however, show an increased expression of vimentin in lens epithelial cells [283]. In a study of 90 patients with congenital cataract, a missense mutation in coil 1B of *VIM* was shown to result in abnormal cell cytoskeletal structure and pulverulent cataracts [2], possibly due to misfolding of vimentin. Another study reported in an ex vivo model of diabetic cataract tissue, the mesenchymal marker vimentin was upregulated while the epithelial marker E-cadherin was downregulated [284]. This same study showed downregulation of the microRNA miR-30. However, the induced overexpression of a variant of this microRNA, miR-30a-5p, decreased vimentin levels, which suggested that miR-30a-5p is a novel therapeutic target for diabetic cataracts. Although not all cataracts are believed to be due to aberrant EMT, these observations indicate that formation of cataracts can arise from EMT transdifferentiation of the cells of the lens epithelium into mesenchymal cells, which thereby cause the cataract opacification [283]. Therefore, repression of EMT regulators might offer a novel means to treat this condition [284].

### 3.10. Crohn’s Disease

Crohn’s disease is a genetic inflammatory bowel disease within the gastrointestinal tract, and is associated with upregulation of vimentin protein levels [285]. The invasive properties of the cells of Crohn’s disease are linked to vimentin expression, as are inflammatory, bacterial, and signalling events [286]. Further studies have shown tissue damage due to inflammation, and the corresponding intestinal fibrosis might be due to EMT [287]. Fibrotic areas show EMT-related markers, and particularly vimentin, which suggests that EMT is involved in the pathogenesis of Crohn’s disease [288]. Moreover, vimentin-targeted treatment of Crohn’s-disease-associated *Escherichia coli* with withaferin-A promotes the correct functioning of the inflammatory response, autophagy, and cell invasion [286].

### 3.11. Rheumatoid Arthritis

The synovial lining acts as the epithelium for joint tissues, and as such it shows similar characteristics. Chronic joint pain associated with rheumatoid arthritis stems from hyperplasia of the tissues surrounding the synovial membrane and cell invasion, a phenomenon that might be due to EMT [289]. In a comparison of biopsies from normal and rheumatoid arthritis diseased tissues, the healthy tissues expressed epithelial-like biomarkers (e.g., E-cadherin, collagen type IV), while the pathological synovium expressed fibrotic markers (e.g., α-smooth muscle actin, vimentin) [289]. Approximately 40% of all sera from patients with rheumatoid arthritis showed autoantibodies directed towards an auto-antigen, known as Sa. This antigen was then shown to be a mutated citrullinated variant of vimentin (MCV) [183]. These anti-MCV antibodies can be detected early in the disease, and anti-MCV titres are closely related to the progress of the disease. Therefore, these data allow for early diagnosis and adequate prognosis of rheumatoid arthritis, and also the evaluation of the therapeutic options [290,291]. Citrullination of vimentin during inflammation has been reported to trigger the antigenic properties within the filament [292] Additional studies have reported that citrullination and mutations of vimentin result in this autoantibody response [293]. These findings show that citrullinated vimentin is an important biomarker for diagnosis and prognosis of rheumatoid arthritis.

### 3.12. Human Immunodeficiency Virus

In a comparative proteomic study, vimentin was recognized as a prospective therapeutic target against HIV [5]. A human dialysable leukocyte extract was shown to regulate vimentin levels and to have anti-HIV activity [5,294]. The vimentin levels and the structure of vimentin were also shown to control the replication of HIV in MT4 cell lines [5]. Together with the findings that the intermediate filament-mimicking synthetic peptide CIGB-210 that causes a reorganisation of vimentin filaments towards the cell nucleus, also inhibits HIV replication [5], these data suggest that vimentin might be a target for anti-HIV treatment.

### 3.13. Atherosclerosis

Endothelial cells can transdifferentiate into mesenchymal-like cells in an analogous manner to EMT of epithelial cells, which is known as endothelial-to-mesenchymal transition (EndMT) [295]. This can cause various diseases of the cardiovascular system, as reviewed by Kovacic and colleagues [296]. For example, EndMT has been observed in atherosclerotic lesions, and has been suggested to be linked to increased vimentin levels [297]. Furthermore, vimentin-null mice show defective capacity to remodel arteries and increased stiffness, contractility and endothelial dysfunction in arteries. Although the increased arterial stiffness in mice lacking vimentin is probably dependent upon endothelial basement membrane reorganization [98], it is possible that vimentin contributes to the phenotypic changes during EndMT.

### 3.14. Defective Wound Healing

Intermediate filaments are crucial for effective wound healing and tissue regeneration through their promotion of cell motility and adhesion. Vimentin has a role in wound healing as it acts as a signal integrator and coordinator for the actions that are necessary: fibroblast proliferation, keratinocyte differentiation, collagen accumulation and re-epithelialisation [104,202].

In a comparative murine model of burn wounds, it was shown that loss of vimentin leads to slow and incomplete healing. In this study, *Vim*−/− epithelial cells inhibited fibroblast growth, which deprives a wound of the collagen that is necessary for healing [104]. Suppression of fibroblasts in turn inhibits TGF-β1 and Slug signalling, which are two of the primary initiators of EMT [104]. TGF-β is known to enhance the migratory behaviour of keratinocytes [298]. In vimentin-null mice, the reduced EMT-like pathways and reduced mobility of keratinocytes result in the loss of keratinisation, and therefore this slows re-epithelialisation. Reconstitution of vimentin in fibroblasts revives the TGF-β–Slug–EMT pathway and reactivates keratinocyte transdifferentiation and migration [104]. As these data show, loss of vimentin leads to slowed and incomplete wound closure, and therefore indicates that vimentin expression is necessary for correct wound healing.

Another study on the regenerative capacity in embryonic and adult mice reported that adult *Vim*−/− mice showed delayed fibroblast migration to a wound site, while the vimentin-deficient embryonic mice failed to heal altogether. Here, they concluded that fibroblasts require vimentin to maintain the tractional forces that are necessary for migration to a wound site [105]. This conclusion is in agreement with a previous ex vivo study in which *Vim*−/− fibroblasts showed slowed migration and collagen contraction, compared to their wild-type counterparts. Delayed wound healing in vimentin-null cells appears to be due to weakened contractile forces from diminished mechanical stability and cell motility [140]. Phosphovimentin-deficient mice show defective wound healing [299], which indicates that the phosphorylation of vimentin is critical for wound healing.

An ex vivo lens model showed that vimentin-rich mesenchymal cells promoted the cell migration for the healing of the wounded epithelium [300]. Similarly, increased rates of migration and wound repair have been associated with overexpression of vimentin in alveolar epithelial cell [201]. Taken together, these studies demonstrate a critical role for vimentin in cellular repair, through regulation of cell movement, adhesion and differentiation in response to wounding.

### 3.15. Vimentin Myopathies

Studies have reported increased expression of vimentin in myopathies, indicating that vimentin can serve as a diagnostic marker for dystrophic muscles and myopathies [301,302,303].

### 3.16. Vimentin in Aging

Vimentin has also been linked to cell senescence, whereby senescent cells have been shown to have increased vimentin mRNA and protein levels, and increased secretion of an oxidised form of vimentin, suggesting that this vimentin variant could be a marker for oxidative stress and aging [304]. It has also been reported that glycation of vimentin in vivo is mainly seen in skin fibroblasts of elderly donors [64]. Furthermore, a lack of serine phosphorylation of vimentin during mitosis results in defective cytokinesis in fibroblasts and lens epithelial cells, and thence to increased senescence [299] and increased skin aging in mice [305]. These data suggest that vimentin also has a role in aging.

**Figure 4:**
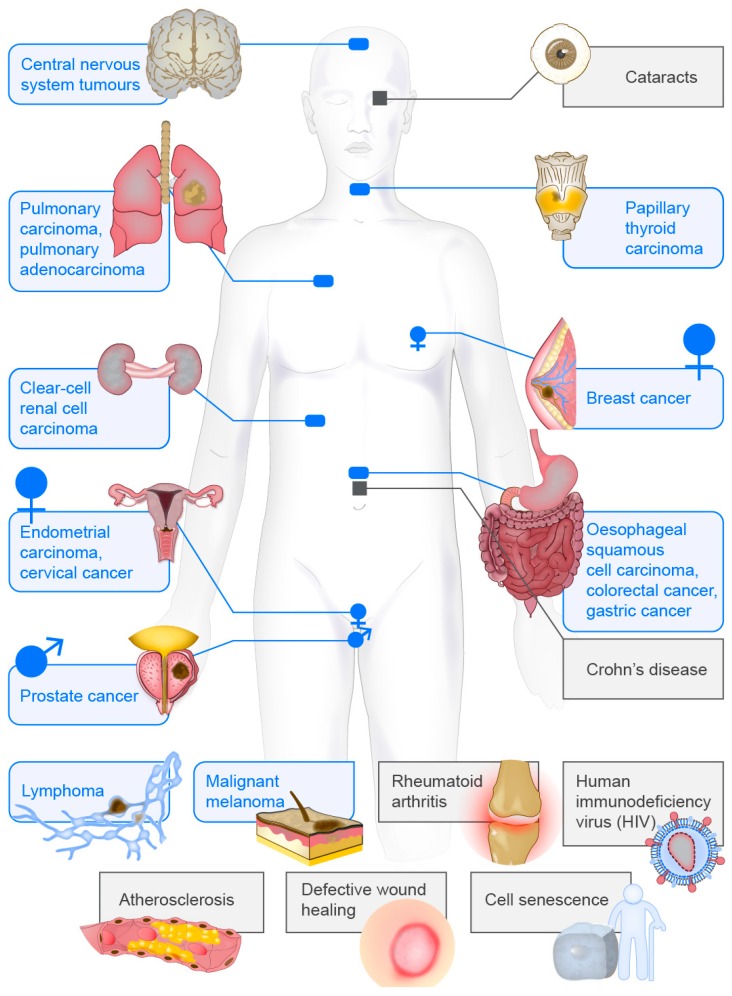
Diseases linked to defective functions of the vimentin protein. Neoplasms are shown in blue boxes, other pathologies in grey boxes.

Taken together, these data indicate that vimentin can be used as a biomarker for diagnosis, prognosis and treatment of a large variety of different diseases, which range from cancer to infectious and inflammatory diseases, which might now allow for more efficient and personalised diagnosis and treatment of these conditions (Figure 4).

### 3.17. Vimentin-Targeting Drugs

Due to its overexpression in many kinds of cancers and its function in tumorigenic events, vimentin is an appealing drug target for cancer therapies. One drug that can interfere with vimentin is withaferin-A, a compound that is known for its anti-tumour and anti-angiogenic activities [306,307]. Withaferin-A has been described to covalently bind to vimentin and result in aggregation of vimentin and F-actin, which leads to cell apoptosis [306]. The apoptotic and antiangiogenic properties of withaferin-A were shown to be significantly reduced in vimentin-deficient cells [308], and withaferin-A treatments have been shown to be beneficial in vivo for patients with breast cancer [309], cervical cancer [310], and pancreatic cancer [311]. In addition, the compound Arylquin 1 (a 3-arylquinoline derivate) has been shown to specifically bind to vimentin, resulting in tumour cell apoptosis via a secretion-dependent mechanism [312]. Also, Silibinin, a compound that has been shown to suppress vimentin expression, reverse EMT and inhibit tumour–stromal communication, also inhibits cancer [313,314,315,316]. In addition, Salinomycin has been reported to suppress vimentin expression [317]. Furthermore, the DNA aptamer NAS-24 has been reported to promote apoptosis in adenocarcinoma cells, potentially by binding to vimentin [318]. Additionally, the vimentin-binding compound, FiVe1 (for FOXC2-inhibiting Vimentin effector 1), has been shown to selectively inhibit the growth of mesenchymally transformed breast cancer cells and soft tissue via mitotic disruption [319].

Another promising avenue for vimentin-targeted treatments was reported by Noh et al. (2016) in their studies of vimentin at the cell surface. They observed that targeting vimentin at the cell surface of glioblastoma stem using a monoclonal antibody, resulted in apoptosis and suppressed proliferation of cells ex vivo, as well as reduced tumour size and increased survival in vivo [320]. Vimentin can have a further role in such treatments by directing therapeutic agents directly to the tumour site. Interleukin-12 is a known cancer therapy, but it has toxic side effects for the patient. However, by binding to the carcinoma cells, the peptide VNTANST can increase the interleukin-12 specifically in the tumour microenvironment. This binding of the peptide to vimentin can be used to better target interleukin-12, which can thus decrease its toxic side effects and increase its direct anticancer effects [321].

Statins are a group of drugs that are known for their cholesterol-lowering properties, as well as being efficient anticancer drugs; these can also target the vimentin intermediate filaments. Simvastatin has been shown to promote cell death in cells expressing vimentin, while not affecting cells that lacked vimentin expression [322]. Furthermore, vimentin has been shown to be a pharmacologically relevant target of Simvastatin in cancer cells [323]. Fluvastatin has also been shown to induce proteolysis of vimentin and to promote cell death in carcinoma cell lines [324]. Of the compounds in this article, these two statin compounds are or have been in clinical trials (U.S. National Library of Medicine, Web-based resource ClinicalTrials.gov).

Taken together, these data indicate that the targeting of vimentin is a promising approach for anticancer drugs.

## 4. Conclusions and Future Perspectives

The aim of this review is to provide an overview of the current understanding of the diversity of vimentin in health and disease, as is also summarised in Figure 2, Figure 3 and Figure 4. However, it is not possible to focus equally on all aspects of vimentin biology, and so all lines of vimentin research cannot be included. The focus was thus on how a single gene product, vimentin, can influence so many cellular functions and diseases.

Vimentin has a highly diverse set of biological functions, both in time and space, and also in terms of the chemical, spatial and mechanical properties of this protein. Several factors are involved in the functional diversity of vimentin. For example, the different domains of the vimentin protein appear to have been subjected to distinct evolutionary pressures, which suggests that separate functions can be performed by specific parts of the vimentin molecule.

The findings that vimentin has many different assembly stages, sizes and conformational forms indicates that its structural plasticity is likely an important factor in the functional diversity of vimentin. In addition, the co-assembly of vimentin with other types of intermediate filaments will increase its functional diversity. Furthermore, cell-type-specific post-translational modifications of vimentin will also change the properties of vimentin. Finally, as different levels of vimentin influence its binding with varying downstream effectors, with different consequences for the signalling pathways involved, both the intracellular and extracellular levels of vimentin are likely to be important for its functional diversity. Further clarification of how these vimentin levels, domains, post-translational modifications and various assembled forms contribute to the functions of vimentin is needed. Given the structural plasticity and functional diversity of the vimentin protein, it is quite plausible that vimentin provides a platform through which it both responds to and integrates biochemical and mechanical signals as well as spatial transduction cues [325].

One of the most repeated phrases in biology is: “Nothing in biology makes sense except in the light of evolution.” This phrase was taken from an essay of the same name published in 1973by Theodosius Dobzhansky, an evolutionary biologist and Eastern Orthodox Christian [33]. It highlights that the process of change is an inherent characteristic of living organisms, which are therefore continuously subjected to, and under the governance of, the laws of evolutionary selection. As a consequence, no biological system can ever be stronger than the diversity that it encompasses. The functional diversity of vimentin could allow it to act as a sensor of altered chemical, spatial and mechanical properties of the cell, and coordinate cellular functions to provide resilience through processes of change. In line with this hypothesis is the observation that the different vimentin functions often are linked to changed cellular behaviour (Figure 3). Cells lacking vimentin appear to be unable to adapt to pathological conditions to the same degree as vimentin-expressing cells. Hence, the increased levels of vimentin that are observed in organisms under a wide variety of pathological conditions might be a consequence of the upregulation of vimentin to provide resilience in a stressed cell, allowing changes that result in pathological cell behaviour.

Future studies to determine how vimentin might control and coordinate the mechanical, spatial and biochemical properties of cells that undergo change are needed. These studies may be aimed at defining vimentin-dependent control of cell surface receptors, localisation of cell adhesions, intracellular biochemical signalling cascades, and localisation of organelles to the relevant spatial and mechanical subcellular areas. Combined with advances in new ‘-omics’ technologies that enable analyses at a single cell level, these studies might ultimately capture the cell-to-cell variability and the role of vimentin in three-dimensional environments. Future studies might examine how vimentin controls the various cellular properties in a more complex tissue context, where different cell types also interact. Such results would advance the understanding of the pathology of vimentin-dependent changes in cell behaviour that result in specific tumour microenvironments, cancers, chronic inflammation, de novo vascularisation, stem cell differentiation and tissue regeneration, as well as defective wound healing.

## Figures and Tables

**Table 1:** Critical in vivo studies highlighting the effects of vimentin knockout in murine animal models, classified according to the cellular effect.

Resulting Phenotype	Reference
*Vim*−/−; no specific disease involvement, *Vim* R113C point mutation; disease phenotype in the eye lens, with increased levels of vimentin aggregates in the eye lens, ultimately leading to posterior cataracts	Bornheim, Müller et al., 2008 [93]
*Vim*−/−; delayed mammary duct growth in adult mice; reduces basal-to-luminal epithelial cell ratio	Peuhu, Virtakoivu et al., 2017 [94]
*Vim*−/−; underdeveloped Bergmann glia cells and Purkinje cells of the cerebellum and motor coordination deficits	Colucci-Guyon, Giménez et al., 1999 [95]
*Vim*−/−; desmin bundles restricted to the perinuclear region of cells	Geerts et al., 2001 [96]
*Vim*−/−; compromised endothelial integrity; defective lymphocyte migration and adhesion to endothelial cells	Nieminen, Henttinen et al., 2006 [97]
*Vim*−/−; reorganisation and increased density of the basement membrane; increased arterial stiffness	Langlois, Belozertseva et al. 2017 [98]
*Vim*−/−; loss of a protective lymphocyte cage; more deformable splenocytes	Brown, Hallam et al., 2001 [99]
*Vim*−/−; increased arterial stiffness and contractility; endothelial dysfunction; no arterial remodelling	Schiffers, Henrion et al., 2000 [100]
*Vim*−/−; disrupted Notch signalling; fewer aortic rings sprouts	Antfolk, Sjöqvist et al., 2017 [101]
*Vim*−/−; 100% lethality when renal mass was decreased by 75%; decreased nitric oxide synthesis, which impaired vasodilation. When treated with the receptor antagonist bosentan proper kidney function maintained	Terzi, Henrion et al., 1997 [102]
*Vim*−/−; decreased gut inflammation and enhanced bacterial killing in acute colitis	Mor-Vaknin, Legendre 2013 [103]
*Vim*−/−; stunted fibroblast growth; slowed reepithelization; slowed and incomplete wound healing	Cheng, Shen et al., 2016 [104]
*Vim*−/−; delayed fibroblast migration to a wound site due to decreased tractional forces; no wound healing	Eckes, Colucci-Guyon et al., 2000 [105]
*Vim*−/−; protection against bacterial meningitis	Huang, Chi et al., 2016 [106]
*Vim*−/−; impaired microglia activation; reduced cerebral ischemia and neurotoxicity	Jiang, Slinn et al., 2012 [107]
*Vim*−/−; no disease phenotype	Colucci-Guyon, Portier et al., 1994 [91]
*Vim*−/−; normal inflammatory response; normal and similar apoptotic rate of lipopolysaccharide-treated neutrophils	Moisan, Chiasson et al., 2007 [108]
*Vim*−/−; halted nestin polymerization in neural stem cells; no increase in apoptosis	Park, Xiang et al., 2010 [109]

**Table 2:** Publications measuring mechanical properties of cells depending on vimentin, classified according to the measurement method. Cells were adhered to an underlying substrate if not described otherwise. An asterisk indicates studies showing that vimentin levels correlate with stiffness.

Methods	Part of Cell Tested	Cell Type	Vimentin Interfering Method	Result of Vimentin Perturbation	Reference
Magnetic bead rheology (rotational force)	Cell cortex	Fibroblasts	*Vim*−/−	* Reduced cell stiffness **Reduced mechanical stability**	Eckes et al., 1998 [140]
Magnetic bead rheology (rotational force)	Cell cortex	Fibroblasts	*Vim*−/−	* Reduced cell stiffness **Reduced cell stiffening after large strains**	Wang and Stamenovic 2000 [141]
Magnetic bead rheology (rotational force)	Cell cortex	Fibroblasts	*Vim*−/−	**No effect**	Guo, Ehrlicher et al., 2013 [124]
Optical tweezer	Cytoplasm	Fibroblasts	*Vim*−/−	* Decreased shear modulus	Guo, Ehrlicher et al., 2013 [124]
Shear Flow	Cell surface	Endothelial cells	No extra vimentin	**Higher variability in vimentin fibre movement**	Helmke, Goldman et al., 2000 [142]
AFM	Cell cortex	Immortalised fibroblasts	Oncogenes increasing total level and soluble fraction of vimentin	* Increased cell stiffness	Rathje, Nordgren et al., 2014 [88]
AFM	Perinuclear region (cytoplasm and cortex)	Fibroblasts	Non-filament-forming desmin mutation; vimentin collapse	* Localized increase of stiffness in perinuclear region of cytoplasm	Plodinec, Loparic et al., 2011 [56]
AFM	Cortex above nucleus	Breast cancer cells	SiRNA, ShRNA Overexpression	* Reduced cell stiffness and impaired mechanical strength **Increased cell stiffness**	Liu, Lin et al., 2015 [143]
Micropost arrays	Whole cell	Breast cancer cells	SiRNA, ShRNA	* Reduced contractile force **Impaired force generation**	Liu, Lin et al., 2015 [143]
Magnetic bead rheology (rotational force) + substrate stretching	Cell cortex	Chondrocytes	Acrylamide	* Reduced cell stiffness **Decreased fluidization‒resolidification response after stretch**	Chen, Yin et al., 2016 [144]
Traction force microscopy	Whole cell	Chondrocytes	Acrylamide	**Reduced traction force after compression**	Chen, Yin et al., 2016 [144]
Image analysis	Nuclear stiffness	Human mesenchymal stem cells		* Stiffening of the nucleus	Keeling, Flores et al., 2017 [145]
Thin film deformation with finite element modelling	Whole cell	Fibroblasts	*Vim*−/−	**Increase in contractile stress (3-fold)**	van Loosdregt et al., 2018 [146]
Agarose-embedded cells (<20% strain)	Whole cell deformation	Mesenchymal stem cells	ShRNA	* Decreased cell deformability	Sharma, Bolten et al., 2017 [147]
Alginate-embedded cells (<20% strain)	Whole cell deformation	Primary human chondrocytes	Acrylamide	* Reduced cell stiffness	Haudenschild, Chen et al., 2011 [148]
Optical stretcher on suspended cells	Whole cell deformation	Natural Killer cells	Withaferin-A	* Increased deformation	Gladilin, Gonzalez et al., 2014 [149]
